# Comparing mid-term outcomes and patient satisfaction between percutaneous endoscopic lumbar discectomy and microendoscopic discectomy for foraminal and extraforaminal lumbar disc herniations: a retrospective matched cohort study

**DOI:** 10.3389/fsurg.2025.1554970

**Published:** 2025-02-19

**Authors:** Sen Liu, Feng Zhao, Chun-Ping Yin, Chao-Hua Zhu, Ruo-Yu Zhao, Guo-Bin Liu, Gang Ji, Jia Chen, Hong-Yang Gao

**Affiliations:** Department of Orthopedics, The First Hospital of Hebei Medical University, Shijiazhuang, China

**Keywords:** PELD, MED, satisfaction rate, foraminal and extraforaminal lumbar disc herniations, inpatient cost assessment

## Abstract

**Objective:**

This study aimed to compare the mid-term outcomes and patient satisfaction between percutaneous endoscopic lumbar discectomy (PELD) and microendoscopic discectomy (MED) for the treatment of foraminal and extraforaminal lumbar disc herniations.

**Methods:**

A retrospective matched cohort study was conducted, including patients diagnosed with foraminal or extraforaminal lumbar disc herniations who underwent PELD or MED between January 2014 and December 2021. Patient demographics, clinical characteristics, and perioperative data were analyzed. Primary outcomes included Visual Analog Scale (VAS) scores for pain, Japanese Orthopaedic Association (JOA) scores and improvement rates for functional status, and overall satisfaction at a minimum 2-year follow-up.

**Results:**

A total of 133 patients were included in the final analysis. The PELD group demonstrated a significantly greater reduction in VAS scores for low back pain (6.74 ± 1.21 to 1.95 ± 0.42) compared to the MED group (6.93 ± 1.17 to 2.35 ± 0.89) at the 2-year follow-up (*p* < 0.001). Both groups exhibited significant improvements in JOA scores, with no notable differences observed at the final follow-up. Patient satisfaction rates were higher in the PELD group, with 86% reporting high satisfaction compared to 72% in the MED group; however, this difference was not statistically significant. Logistic regression analysis identified VAS scores for low back pain, operation cost, and symptom recurrence as independent factors influencing patient dissatisfaction at 2 years post-surgery.

**Conclusion:**

Both PELD and MED demonstrated efficacy in treating foraminal and extraforaminal lumbar disc herniations over a 2-year follow-up period. PELD, however, exhibited superior relief of low back pain. Factors, such as low back pain intensity, surgical costs, and symptom recurrence significantly impacted patient dissatisfaction, despite comparable overall satisfaction rates between the two surgical techniques.

## Introduction

Lumbar disc herniation is a prevalent condition that significantly impacts individuals by causing severe pain, disability, and a reduced quality of life (1). Among the various types of lumbar disc herniations, foraminal and extraforaminal herniations impose unique diagnostic and treatment challenges due to their anatomical location and the potential for nerve root compression (2, 3). Abdullah et al. (1974) first described the clinical syndrome of far lateral (extraforaminal) lumbar disc herniation (4). This condition arises from the protrusion of the intervertebral disc into the foraminal or extraforaminal regions, leading to mechanical nerve root compression or inflammation at the affected level. Mechanical compression typically occurs due to direct pressure on the nerve root by the herniated disc material, often exacerbated by a narrow foraminal space or degenerative changes, such as osteophyte formation. Additionally, inflammation plays a crucial role in symptom development, as the extruded disc material contains pro-inflammatory mediators, including tumor necrosis factor-alpha (TNF-α), interleukin-1 beta (IL-1β), and prostaglandins. These mediators can induce a cascade of inflammatory responses, causing localized edema, increased vascular permeability, and sensitization of nociceptors. Furthermore, anatomical variations, such as the location of the dorsal root ganglion in close proximity to the extraforaminal space, make these regions particularly susceptible to severe and persistent radicular pain. These mechanical and inflammatory factors contribute to the complex clinical presentation and therapeutic challenges associated with foraminal and extraforaminal lumbar disc herniations.

Clinical series reported an incidence rate of far lateral lumbar disc herniation ranging from 1% to 12% of observed or surgically treated cases of lumbar disc herniation. These cases are typically characterized by nerve root compression at the dorsal ganglia, often manifesting as significant leg pain, whereas back pain is generally mild to moderate. Compression involving the dorsal ganglia frequently leads to a more severe and complex pain syndrome compared to intraforaminal or intracanal disc herniations.

Traditional open surgical techniques have long been employed for managing these cases. However, advances in minimally invasive procedures have introduced alternative surgical options, including percutaneous endoscopic lumbar discectomy (PELD) and microendoscopic discectomy (MED) (5, 6). PELD, distinguished by its minimally invasive approach, offers several benefits, such as reduced tissue disruption, shorter recovery periods, and fewer postoperative complications (7–9). Similarly, MED employs an endoscope to provide improved visualization of the surgical field, enabling a minimally invasive approach with comparable benefits (10).

While PELD and MED have gained traction as minimally invasive alternatives to traditional open surgery, existing research on their efficacy presents limitations. Several studies have concentrated on outcomes for general lumbar disc herniations, while provided insufficient evidence on their effectiveness specifically for foraminal and extraforaminal herniations, resulting in unique anatomical and surgical challenges. For instance, PELD has been associated with advantages, such as reduced tissue trauma and shorter recovery time (7–9); however, some reports highlighted limitations, such as a steeper learning curve, restricted surgical field visualization, and potential difficulty accessing extraforaminal regions in certain cases (11, 12). Similarly, while MED provides enhanced visualization and precision, it may be associated with increased intraoperative complexity and a higher risk of dural tears in comparison to PELD (13, 14). These drawbacks reflect the need for more comprehensive analyses to determine the relative advantages of these techniques in managing foraminal and extraforaminal herniations. Furthermore, the mid- to long-term outcomes of these approaches remain inadequately explored, with most studies concentrating on short-term recovery metrics. This gap in the literature necessitates further research to provide clinicians with evidence-based guidance tailored to the unique challenges posed by these herniations.

Despite the increasing adoption of minimally invasive techniques, comprehensive evaluations comparing mid-term outcomes of PELD and MED for foraminal and extraforaminal lumbar disc herniations remain limited. This study seeks to address this gap by performing a retrospective matched cohort analysis to compare the 2-year clinical outcomes of PELD and MED in patients with these specific types of herniations. The findings aim to provide clinicians with evidence-based insights to guide treatment decisions and contribute to the refinement of surgical management strategies for this challenging condition.

## Methods

### Study design

This study employed a retrospective matched cohort design to compare the 2-year outcomes of PELD and MED in patients diagnosed with foraminal and extraforaminal lumbar disc herniations. All procedures were performed by experienced spine surgeons between January 2014 and January 2021.

### Ethical considerations

The study adhered to the principles of the Declaration of Helsinki and received approval from the institutional review board (IRB) of the First Hospital of Hebei Medical University. Informed consent was obtained from all participants prior to their inclusion in the study.

### Patient selection

Patients were identified through a comprehensive review of surgical databases. The inclusion criteria were adult patients (aged 18–65 years) who underwent either PELD or MED for confirmed foraminal or extraforaminal lumbar disc herniations, as verified by MRI. Exclusion criteria included a history of prior spinal surgery, significant comorbidities that could affect recovery, and insufficient follow-up data at the 2-year mark.

### Matched cohort design

Patients were matched using a systematic approach to ensure comparability between the PELD and MED groups. Matching was performed based on key demographic and clinical variables, including age, gender, body mass index (BMI), and preoperative clinical presentation (pain severity and functional status, assessed by Visual Analog Scale and Japanese Orthopaedic Association scores). A 1:1 matching ratio was applied to minimize confounding factors, and propensity score matching was utilized to enhance the accuracy of the matching process. The propensity scores were calculated using a logistic regression model, with the treatment type (PELD or MED) as the dependent variable and the selected baseline characteristics as independent variables. A caliper width of 0.2 standard deviations of the logit of the propensity score was used to ensure close matches while retaining sufficient sample size. This method was chosen to reduce selection bias and achieve a balance between the groups. The inclusion of these specific matching variables was justified by their relevance to patient outcomes and potential influence on treatment effectiveness. Variables such as age and BMI were included due to their established associations with surgical recovery, while pain severity and functional status were critical for evaluating baseline comparability in clinical presentation.

### Surgical techniques

The surgical techniques used were as follows:

PELD: The PELD procedure was performed using a unilateral approach under general anesthesia with sedation. A working cannula was inserted, and endoscopic visualization facilitated the removal of herniated disc material while preserving surrounding structures.

MED: The MED procedure employed a minimally invasive approach utilizing a microendoscope. An incision was made to provide direct visualization for the removal of herniated disc fragments. This procedure was conducted under general anesthesia.

To ensure consistency in surgical procedures, all PELD and MED operations followed standardized protocols established by the institution. These protocols included predefined guidelines for patient positioning, instrument selection, and procedural steps. Specific surgical instruments were uniformly employed for each procedure type to minimize variability. Additionally, all surgeries were performed by a team of experienced spine surgeons, each trained in the respective techniques, to reduce inter-surgeon variability. Regular peer reviews of surgical approaches were conducted to ensure adherence to these standards. However, minor variations tailored to individual patient anatomy and pathology were documented and accounted for in the analysis.

### Outcome measures

The efficacy of the surgical interventions for lumbar disc herniation was evaluated both preoperatively and at a 2-year follow-up, focusing on mid-term success rates and patient recovery. The primary outcome measures included were as follows:

1) Pain Level: Pain was assessed using the Visual Analog Scale (VAS), where patients rated their pain on a scale from 0 (no pain) to 10 (worst imaginable pain).
2) Functional Improvement: The Japanese Orthopaedic Association (JOA) score was used to assess functionality and pain levels. The scores range from a maximum of 29 points to a minimum of 0 points, with lower scores indicating greater dysfunction. The Improvement Rate was calculated using the following formula:
  ImprovementRate=(PostoperativeScore-PreoperativeScore)/(29-PreoperativeScore)×100%

Improvement rate was categorized into different categories based on percentage of the improvement. 100%, >60%, 25%–60%, and <25% were classified as a cure, marked effectiveness, effectiveness, and ineffectiveness, respectively.

In addition to pain level (VAS), functional improvement (JOA), and patient satisfaction index (PSI), other outcomes, such as complication rates, reoperation rates, and return-to-work status were considered during the study design. However, due to limitations in the retrospective nature of the study and inconsistent documentation, these metrics were not systematically collected and analyzed. Future studies will incorporate these additional outcomes to provide a more comprehensive evaluation of surgical efficacy and long-term recovery.

### PSI

To minimize bias, data collection for surgical procedures and research variables was conducted by different personnel. Before surgery, patients completed an initial questionnaire regarding demographic information. The PSI was used to evaluate self-assessed outcomes (11). Responses of 1 or 2 were classified as satisfactory, while responses of 3 or 4 were deemed unsatisfactory (Table 1).

**Table 1:** Patient satisfaction index.

PSI score	Description
1 Very satisfied	Surgery met my expectations.
2 Satisfied	Surgery improved my condition enough so that I would undergo it again for the same outcome.
3 Dissatisfied	Surgery helped me, but I would not go through it again for the same outcome.
4 Very dissatisfied	I am the same or worse compared to before surgery.

At the final follow-up, researchers conducted telephone surveys to collect PSI data and analyze its relationship with patient satisfaction and functionality. Patients were divided into satisfied and unsatisfied groups based on their PSI responses.

### Hospitalization cost assessment

Hospitalization costs were collected from the hospital's electronic medical records at the final follow-up and included direct inpatient expenses such as drugs, diagnostic examinations, surgical procedures, nursing care, and postoperative rehabilitation. The costs were calculated in the local currency and were subsequently adjusted for inflation using the official Consumer Price Index (CPI) data for the study period (2014–2021) to ensure comparability over time. Additionally, regional economic differences were considered by normalizing costs against the average per capita income of the province during the respective year. Insurance coverage variations were accounted for by stratifying patients based on their insurance type (e.g., public, private, or uninsured) and evaluating the out-of-pocket expenses separately. These adjustments aimed to provide a standardized and comprehensive analysis of the economic burden associated with the treatments (12).

### Statistical analysis

Statistical analyses were conducted using SPSS version 21.1. Continuous variables were summarized using descriptive statistics, while differences between groups were analyzed using independent *t*-tests for parametric data and Chi-square tests for categorical variables. Survivorship curves were compared using log-rank tests to identify significant differences. A *p*-value of <0.05 was considered statistically significant for all analyses.

## Results

In this retrospective matched cohort study, 133 patients were analyzed, with 78 undergoing PELD and 55 undergoing MED. Baseline demographics and clinical characteristics, including age, sex, preoperative status, JOA scores, and VAS scores for lower back pain (LBP) and leg pain, were comparable between the two groups, ensuring a reliable comparison of treatment outcomes (Table 2).

**Table 2:** The main demographic variables of patients before surgery.

Items	PELD group (*n* = 78)	MED group (*n* = 55)	*p*-value
Age (years)	46.91 ± 5.12	47.92 ± 5.54	0.283
Sex ratio (M/F)	37/41	22/32	0.447
BMI (kg/m^2^)	23.28 ± 3.11	23.90 ± 2.94	0.254
Smoking (Yes/No)	21/57	14/41	0.850
Drinking (Yes/No)	17/61	12/33	0.540
Heart disease (Yes/No)	10/68	10/45	0.394
Hypertension (Yes/No)	12/66	6/49	0.457
Diabetes (Yes/No)	9/69	6/49	0.910
Segment of lesion			0.260
L3–4	17	6	
L4–5	39	32	
L5–S1	22	17	

*p* < 0.05 indicates a statistically significant difference between the two groups; PELD, percutaneous endoscopic lumbar discectomy; MED, microendoscopic discectomy.

### Pain relief

Both PELD and MED resulted in significant reductions in pain levels at the 2-year follow-up, as measured by the VAS. The PELD group showed a marked decrease in VAS scores for lower back pain, from 6.74 ± 1.21 to 1.95 ± 0.42, which was significantly greater than the reduction observed in the MED group, where scores declined from 6.93 ± 1.17 to 2.35 ± 0.89 over the same period (*p* < 0.001) (Table 3).

**Table 3:** Clinical outcomes between the two groups.

Items	PELD group (*n* = 78)	MED group (*n* = 55)	*p*-value
Operation time (min)	70.27 ± 11.77	69.43 ± 12.82	0.699
Incision length (mm)	9.91 ± 1.03	20.27 ± 1.66	<0.001*
Blood loss (ml)	49.51 ± 5.60	80.35 ± 6.24	<0.001*
Time of weight-bearing (day)	1.90 ± 0.35	1.95 ± 0.34	0.388
Operation cost (thousand RMB)	20.37 ± 3.25	16.31 ± 3.92	<0.001*
JOA score			
Preoperative	16.01 ± 1.82	15.67 ± 2.52	0.368
Final follow-up	25.42 ± 2.32	25.25 ± 2.14	0.671
*p*-value	<0.001*	<0.001*	
Improvement rate (%)	71.9 ± 18.9	71.4 ± 16.4	0.853
VAS score-lower back pain			
Preoperative	6.74 ± 1.21	6.93 ± 1.17	0.384
Final follow-up	1.95 ± 0.42	2.35 ± 0.89	0.001*
*p*-value	<0.001*	<0.001*	
VAS score-leg pain			
Preoperative	6.74 ± 0.76	6.71 ± 0.88	0.810
Final follow-up	1.74 ± 0.67	1.84 ± 0.37	0.224
*p*-value	<0.001*	<0.001*	
Symptom persist or recurrence	12/66	9/46	0.879
Intermittent pain	8	5	
Persistent pain	4	4	
Postoperative depression (Yes/no)	8/70	7/48	0.657
Satisfaction (Yes/No)	64/14	40/15	0.200
Satisfaction rate	82.1%	72.7%	

PELD, percutaneous endoscopic lumbar discectomy; MED, microendoscopic discectomy.

* Indicates a statistically significant difference.

### Functional improvement

**Figure 1:**
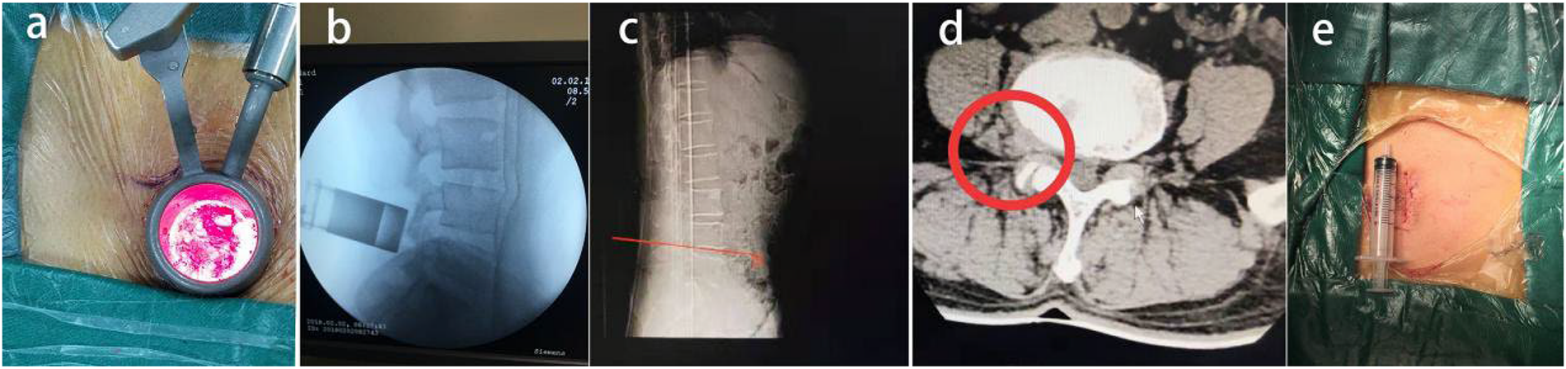
A 43-year-old patient with L4–5 extraforaminal lumbar disc herniation **(a–d)** underwent microendoscopic discectomy treatment with a small incision **(e)**, and the postoperative effect was satisfactory.

Functional outcomes were assessed using JOA scores, which showed significant improvement in both groups. In the PELD group, JOA scores increased from 16.01 ± 1.82 preoperatively to 25.42 ± 2.32 (*p* < 0.001), while in the MED group, scores improved from 15.67 ± 2.52 to 25.25 ± 2.14 (*p* < 0.001). At the final follow-up, no significant difference was observed between the two groups (Figure 1). The JOA improvement rate demonstrated marked effectiveness, with rates of 71.9% in the PELD group and 71.4% in the MED group. However, similar to the JOA scores, the difference between the groups was not statistically significant (*p* = 0.853) (Table 3).

### Patient satisfaction

Patient satisfaction was evaluated through a questionnaire administered at the 2-year follow-up, with responses categorized as very satisfied, satisfied, dissatisfied, and very dissatisfied. Satisfaction rates were higher in the PELD group, with 86% reporting high satisfaction compared to 72% in the MED group. However, no statistically significant difference was found between the groups. Logistic regression analysis revealed that VAS scores for LBP were independently associated with patient dissatisfaction, with an odds ratio (OR) of 0.150 (*p* = 0.001). Additionally, operation cost (OR = 0.816, *p* = 0.005) and symptom recurrence (OR = 8.696, *p* < 0.001) were also significant factors contributing to dissatisfaction 2 years after surgery (Tables 4, 5).

**Table 4:** The related risk factors of satisfied and dissatisfied patients at the 2 your follow-up.

Items	Dissatisfaction (*n* = 29)	Satisfaction (*n* = 104)	*p*-value
Age (years)	48.54 ± 5.66	46.99 ± 5.18	0.166
Sex ratio (M/F)	15/14	44/60	0.367
BMI (kg/m^2^)	23.16 ± 3.74	23.64 ± 2.84	0.454
Operation time (min)	70.14 ± 13.44	69.87 ± 11.87	0.916
Incision length (mm)	1.56 ± 0.55	1.38 ± 0.52	0.119
Blood loss (ml)	63.38 ± 16.47	61.95 ± 16.35	0.679
Time of weight-bearing (day)	1.97 ± 0.35	1.91 ± 0.34	0.374
Operation cost (thousand RMB)	20.38 ± 4.54	18.22 ± 3.08	0.011*
JOA score at final follow-up	25.55 ± 1.82	25.30 ± 2.35	0.592
Improvement rate (%)	74.4 ± 12.4	71.0 ± 18.8	0.399
VAS score-lower back pain at final follow-up	2.66 ± 1.17	1.96 ± 0.34	<0.001*
VAS score-leg pain at final follow-up	1.76 ± 0.44	1.79 ± 0.43	0.744
Symptom recurrence	11/18	10/94	<0.001*

* Indicates a statistically significant difference.

**Table 5:** Binary logistic regression analysis of postoperative dissatisfaction.

Items	*B*	OR	*p* value	95% CI for OR
Hospitalization cost (thousand RMB)	−0.204	0.816	0.005*	(0.708, 0.939)
VAS score-low back pain	−1.898	0.150	0.001*	(0.051, 0.443)
Symptom recurrence (Yes/No)	2.163	8.696	<0.001*	(2.605, 29.021)

95% CI, 95% confidence interval.

* Indicates a statistically significant difference.

Satisfaction rates were influenced by several factors, including postoperative pain levels, symptom recurrence, and operation costs. Subgroup analysis revealed that patients with higher postoperative VAS scores for lower back pain were significantly more likely to report dissatisfaction (*p* < 0.001). Additionally, dissatisfaction was more prevalent in patients who experienced symptom recurrence (*p* < 0.001) or incurred higher operation costs (*p* = 0.011). To further explore socioeconomic influences on satisfaction, factors, such as insurance type, employment status, and income level were examined. While these variables were not systematically collected for all patients, qualitative trends indicated that patients with limited insurance coverage or higher out-of-pocket expenses were less likely to report high satisfaction. Future prospective studies will incorporate detailed socioeconomic data to better elucidate their role in patient satisfaction. The analysis indicated the multifaceted nature of patient satisfaction, which is influenced not only by clinical outcomes, but also by financial burden and recurrence of symptoms. These findings highlight the importance of addressing both medical and socioeconomic factors in the comprehensive evaluation of surgical success.

## Discussion

In 1997, MED was first introduced as a minimally invasive procedure, utilizing advanced microscope technology and optical techniques via an intramuscular approach (13). Over the past few decades, endoscopic techniques have evolved, enabling direct visualization and more localized management during discectomy procedures. Kambin was the first to report the intraoperative endoscopic examination of the disc, and since October 1988, surgical advancements have progressively refined these techniques. In 1997, Yang and Zou developed the Yang endoscopic spinal system (14), while Hoogland et al. (1994) introduced the Thomas Hoogland endoscopic spinal system (15). PELD involves both the transforaminal approach (percutaneous endoscopic transforaminal discectomy, PETD) and the interlaminar approach (percutaneous endoscopic interlaminar discectomy, PEID) (16). Although numerous studies have compared the short-term outcomes of PETD and MED, both exhibiting satisfactory results for treating lumbar disc herniation, there is a notable lack of research concentrating on foraminal and extraforaminal lumbar disc herniations, as well as patient satisfaction surveys.

The present study aimed to compare the mid-term outcomes of PELD and MED in a matched cohort of 133 patients. The findings suggested that both PELD and MED were effective surgical options for pain relief and functional improvement in patients with lumbar disc herniations after a 2-year follow-up. Both techniques were effective in treating lumbar disc herniation, with PELD offering advantages such as smaller incisions, reduced blood loss, and faster recovery, while MED provided benefits like shorter operation times and less x-ray exposure. This finding is significant for clinical practice, as it suggests that patients can choose either technique based on surgeon expertise or patient preference without compromising functional outcomes. However, PTED showed no significant differences compared to MED in terms of functional disabilities and improvement rates. These results align with the findings of a comparative retrospective study by Sinkeman et al. (17), which suggested that PTED and MED can achieve equivalent and satisfactory outcomes.

The findings further confirmed that patients with lumbar disc herniation who underwent PELD experienced positive outcomes, with success rates consistent with the general efficacy of surgical interventions for this condition. It is widely believed that PELD may have comparable or even superior clinical effects to open discectomy in selected patients (18–20). Previous literature has shown that Yeung's endoscopic spine system (YESS) is particularly suitable for treating intraforaminal or extraforaminal disc herniations (21, 22). Our results further validate these earlier findings, especially due to the precision with which the working cannula can be positioned during surgery in the intervertebral foraminal and far-lateral disc herniation regions.

PELD is a demanding technique for treating intervertebral foraminal and far-lateral disc herniation. As an effective minimally invasive surgery, PELD has shown excellent clinical results for this specific type of disc herniation, with a high success rate of over 90% and a low complication rate. It is particularly beneficial for patients who have not responded to conservative treatments, offering a less invasive alternative that preserves spinal integrity and stability. Liu et al. suggested that MED can effectively manage disc herniation by allowing the dura to retract inward, clearly exposing the protrusion. However, due to limitations such as the articular processes and small working cannulas, it can be challenging to reach the ventral aspect of the disc at the foraminal level (23).

Previously, PTED was considered difficult at the L5–S1 level due to anatomical restrictions, such as the high iliac crest (24, 25). However, advancements in technique have made L5–S1 lesions no longer a relative contraindication for PTED. Our results show that PTED at the L5–S1 level yields clinical outcomes equivalent to those at the L4–5 level. In cases involving a high iliac crest, the TESSYS technique offers a method to access the L5–S1 intervertebral foraminal and far-lateral regions through the intervertebral foraminal approach. A 1-year follow-up study by Chen found that the two surgeries had similar safety and effectiveness, as evidenced by LDH. However, the results did not show a clear advantage of PTED over MED in terms of functional disability, back pain, leg pain, and quality of life (26).

While our results showed no significant differences in improvement between MED and PELD, PELD demonstrated superior postoperative pain relief, particularly for lower back pain. Both surgical methods led to substantial reductions in pain levels, as measured by the VAS. Unlike PELD, which directly exposes the protruded area, MED does not offer a wide direct surgical view and often requires extensive bone resection (including facet joints) to properly expose the protruding disc. As a result, insufficient bone volume, particularly damage to the facet joint, may lead to segmental instability postoperatively, contributing to chronic LBP (27). Furthermore, nerve root ganglion damage caused by direct compression and far-lateral disc herniation can impair neural recovery, serving as another factor contributing to postoperative pain (28, 29). In contrast, PTED can directly remove protruded discs within and outside the intervertebral foraminal spaces without compromising the posterior column structure, thus reducing the occurrence of postoperative LBP compared to MED.

Patient satisfaction, a critical indicator of treatment success, was remarkable in both cohorts. The proportion of patients reporting “very satisfied” or “satisfied” responses was 82.1% in the PELD group and 72.7% in the MED group, and no statistically significant difference was identified between the two groups. These findings suggest that both procedures are generally well-received and contribute positively to patients' overall quality of life post-surgery. Satisfaction levels appeared to be influenced by factors, such as preoperative expectations, the postoperative recovery experience, and the perceived effectiveness of the treatment. Notably, a considerable number of patients expressed satisfaction with their surgical outcomes and indicated a willingness to undergo the procedure again. However, dissatisfaction was found in 14 (17.9%) patients from the PELD group and 15 (27.3%) patients from the MED group, as determined by the PSI assessment. This aligns with previous findings by Sinkeman et al., who reported that PTED and MED deliver comparable and satisfactory outcomes (30). Similarly, Wang et al. documented a 91.99% satisfaction rate in a retrospective study of 337 PELD patients (31). Notably, in this study, the presence of a positive Lasegue sign was more common among satisfied patients, possibly due to the correlation with higher preoperative VAS scores. Conversely, dissatisfaction was more frequent in patients with consecutive double-segment intervertebral disc herniations, aligning with the satisfaction trends observed in the current research.

The higher cost of endoscopic instruments made PTED significantly more expensive in terms of surgical and hospitalization costs compared with MED. Despite this, satisfaction rates did not significantly differ between the groups. Both procedures achieved substantial reductions in pain levels, as measured by VAS scores. The analysis highlighted postoperative pain and symptom recurrence as key contributors to patient dissatisfaction. Univariate regression analysis confirmed that postoperative pain independently influenced satisfaction outcomes in both groups, highlighting the importance of effective pain management strategies during the recovery period. Research has demonstrated that targeted pain management protocols and comprehensive nursing interventions can significantly reduce postoperative pain, thereby enhancing patient satisfaction and improving overall outcomes.

Future studies should assess the underlying causes of postoperative pain in spinal surgery patients and explore targeted interventions to address these factors. Additionally, research into the long-term effects of different surgical techniques on pain relief and satisfaction outcomes may provide valuable insights for clinical practice and help optimize patient care. Postoperative pain not only affects patients physically and psychologically, but also increases the likelihood of repeat surgeries, contributing to higher healthcare costs. Such pain can cause distress, complicate treatment adherence, and necessitate additional medications, rehabilitation, and extended care. Patients with lower income and education levels may face additional challenges in understanding their condition and navigating treatment options. Limited education can hinder the ability to critically assess medical information, leaving these patients vulnerable to misinformation from unregulated sources. In some cases, institutions may exaggerate treatment benefits to maximize profits, which can create confusion and erode trust in medical treatments. Blind acceptance of unverified information exacerbates these issues, potentially leading to dissatisfaction and skepticism regarding surgical outcomes.

There is a growing acknowledgment of the influence of economic pressures on patients' satisfaction with spinal surgery outcomes (32, 33). Realistic expectations surrounding surgery are recognized as a critical factor in improving patient satisfaction. Compared with higher-income patients, those from lower-income backgrounds tend to have more optimistic expectations regarding postoperative pain and recovery, even when surgeons provide comprehensive information about potential postoperative complications. Regardless of the severity of their condition, patients prioritize practical outcomes that enable their return to work over theoretical knowledge. This is reflected in the efficacy of rehabilitation programs and the emphasis on functional recovery. Additionally, emotional factors, combined with surgical experiences, play a remarkable role in shaping patient perceptions and contribute to increased treatment costs.

The higher cost of PELD may significantly influence patient decision-making, especially in low-income populations. Financial constraints can lead patients to favor more affordable procedures, such as MED, even when PELD may provide superior long-term outcomes, such as better pain relief and reduced risk of complications, such as chronic lower back pain. For healthcare systems in resource-limited settings, the higher cost of PELD may act as a barrier to widespread adoption, despite its potential for improved clinical outcomes. The substantial cost of endoscopic equipment and the need for specialized training may discourage healthcare providers from offering PELD, limiting access for patients who can benefit from this technique. To enhance access to PELD, policy adjustments are necessary, such as increasing insurance coverage, providing subsidies for the required equipment, or exploring more cost-effective technological innovations. Such measures can help make this procedure more accessible to a wider patient population, particularly in low-income and underserved areas. Further research into the cost-effectiveness of PELD, particularly in low-income populations, is critical. Understanding the long-term economic benefits, such as reduced complication rates and fewer repeat surgeries, can provide valuable evidence to support policy changes that ensure equitable access to the most effective treatment options.

This study has some limitations that should be acknowledged. As a retrospective analysis, there is potential for selection bias, and no randomization was performed. Additionally, factors, such as variations in surgical technique, surgeon experience, and patient adherence to rehabilitation protocols might influence the outcomes. Future prospective, randomized trials with larger sample sizes and extended follow-up periods are necessary to validate these findings and improve their generalizability.

## Conclusion

Although both PELD and MED demonstrate significant effectiveness in treating foraminal and extraforaminal lumbar disc herniations over a 2-year follow-up period, PELD provided superior relief of low back pain. Patient dissatisfaction was associated with low back pain, operation cost, and symptom recurrence, despite similar overall satisfaction rates between the two surgical techniques.

## Data Availability

The datasets presented in this study can be found in online repositories. The names of the repository/repositories and accession number(s) can be found in the article/Supplementary Material.

## References

[1] Liu C Ferreira GE Abdel Shaheed C Chen Q Harris IA Bailey CS Surgical versus non-surgical treatment for sciatica: systematic review and meta-analysis of randomised controlled trials. Br Med J. (2023) 381:e070730. 10.1136/bmj-2022-070730 37076169 PMC10498296

[2] Shawky Abdelgawaad A Babic D Siam AE Ezzati A. Extraforaminal microscopic assisted percutaneous nucleotomy for foraminal and extraforaminal lumbar disc herniations. Spine J. (2018) 18(4):620–5. 10.1016/j.spinee.2017.08.258 28882526

[3] Wang H Sun W Fu D Shen Y Chen YY Wang LL. Update on biomaterials for prevention of epidural adhesion after lumbar laminectomy. J Orthop Translat. (2018) 13:41–9. 10.1016/j.jot.2018.02.001 29662790 PMC5892378

[4] Abdullah AF Ditto EW 3rd Byrd EB Williams R. Extreme-lateral lumbar disc herniations. Clinical syndrome and special problems of diagnosis. J Neurosurg. (1974) 41(2):229–34. 10.3171/jns.1974.41.2.0229 4841878

[5] Tang T Liu J Cao J He D Cheng X Xie S. Risk factors and causes of reoperation in lumbar disc herniation patients after percutaneous endoscopic lumbar discectomy: a retrospective case series with a minimum 2-year follow-up. Med Sci Monit. (2023) 29:e939844. 10.12659/MSM.939844 37580900 PMC10439676

[6] Wei FL Li T Gao QY Yang Y Gao HR Qian JX Eight surgical interventions for lumbar disc herniation: a network meta-analysis on complications. Front Surg. (2021) 8:679142. 10.3389/fsurg.2021.679142 34355013 PMC8329383

[7] Pan M Li Q Li S Mao H Meng B Zhou F Percutaneous endoscopic lumbar discectomy: indications and complications. Pain Physician. (2020) 23(1):49–56. 32013278

[8] Gadjradj PS Harhangi BS Amelink J van Susante J Kamper S van Tulder M Percutaneous transforaminal endoscopic discectomy versus open microdiscectomy for lumbar disc herniation: a systematic review and meta-analysis. Spine. (2021) 46(8):538–49. 10.1097/BRS.0000000000003843 33290374 PMC7993912

[9] Ruetten S Komp M Godolias G. An extreme lateral access for the surgery of lumbar disc herniations inside the spinal canal using the full-endoscopic uniportal transforaminal approach-technique and prospective results of 463 patients. Spine. (2005) 30(22):2570–8. 10.1097/01.brs.0000186327.21435.cc 16284597

[10] Jia H Zhang Z Qin J Bao L Ao J Qian H. Management for degenerative lumbar spondylolisthesis: a network meta-analysis and systematic review basing on randomized controlled trials. Int J Surg. (2024) 110(5):3050–9. 10.1097/JS9.0000000000001929 38446872 PMC11093486

[11] Wang H Zhang D Ma L Shen Y Ding W. Factors predicting patient dissatisfaction 2 years after discectomy for lumbar disc herniation in a Chinese older cohort: a prospective study of 843 cases at a single institution. Medicine. (2015) 94(40):e1584. 10.1097/MD.0000000000001584 26448005 PMC4616769

[12] Liu S Yang SD Fan XW Yang DL Ma L Sun JY Analyses of effect factors associated with the postoperative dissatisfaction of patients undergoing open-door laminoplasty for cervical OPLL: a retrospective cohort study. J Orthop Surg Res. (2019) 14(1):161. 10.1186/s13018-019-1208-8 31138291 PMC6540572

[13] Perez-Cruet MJ Foley KT Isaacs RE Rice-Wyllie L Wellington R Smith MM Microendoscopic lumbar discectomy: technical note. Neurosurgery. (2002) 51(5 Suppl):S129–36. 12234440

[14] Yeung AT Tsou PM. Posterolateral endoscopic excision for lumbar disc herniation: surgical technique, outcome, and complications in 307 consecutive cases. Spine. (2002) 27(7):722–31. 10.1097/00007632-200204010-00009 11923665

[15] Hoogland T van den Brekel-Dijkstra K Schubert M Miklitz B. Endoscopic transforaminal discectomy for recurrent lumbar disc herniation: a prospective, cohort evaluation of 262 consecutive cases. Spine. (2008) 33(9):973–8. 10.1097/BRS.0b013e31816c8ade 18427318

[16] Meng H Su N Lin J Fei Q. Comparative efficacy of unilateral biportal endoscopy and micro-endoscopic discectomy in the treatment of degenerative lumbar spinal stenosis: a systematic review and meta-analysis. J Orthop Surg Res. (2023) 18(1):814. 10.1186/s13018-023-04322-2 37907922 PMC10619222

[17] Smith N Masters J Jensen C Khan A Sprowson A. Systematic review of microendoscopic discectomy for lumbar disc herniation. Eur Spine J. (2013) 22(11):2458–65. 10.1007/s00586-013-2848-8 23793558 PMC3886516

[18] Gadjradj PS van Tulder MW Dirven CM Peul WC Harhangi BS. Clinical outcomes after percutaneous transforaminal endoscopic discectomy for lumbar disc herniation: a prospective case series. Neurosurg Focus. (2016) 40(2):E3. 10.3171/2015.10.FOCUS15484 26828884

[19] Feng Z Wu Y Wu H Jon TG Yuan Y Chen Z A modified laminotomy for interlaminar endoscopic lumbar discectomy: technical report and preliminary results. Neurospine. (2023) 20(4):1513–23. 10.14245/ns.2346572.286 38171317 PMC10762391

[20] Ma H Shen M Tang Z Li K Zhou H Sun X Percutaneous endoscopic interlaminar discectomy for high-grade migrated lumbar disc herniation: clinical efficacy and safety assessment. Int Orthop. (2024) 48(9):2455–63. 10.1007/s00264-024-06246-w 38969821

[21] Spallone A Khalepa RV Amelina E Asif Ogly AM. Endoscopic lumbar disc surgery experience with the TESSYS technique in 253 case series. J Clin Med. (2024) 13(7):1911. 10.3390/jcm13071911 38610676 PMC11012553

[22] Xin G Shi-Sheng H Hai-Long Z. Morphometric analysis of the YESS and TESSYS techniques of percutaneous transforaminal endoscopic lumbar discectomy. Clin Anat. (2013) 26(6):728–34. 10.1002/ca.22286 23824995

[23] Liu X Yuan S Tian Y Wang L Gong L Zheng Y Comparison of percutaneous endoscopic transforaminal discectomy, microendoscopic discectomy, and microdiscectomy for symptomatic lumbar disc herniation: minimum 2-year follow-up results. J Neurosurg Spine. (2018) 28(3):317–25. 10.3171/2017.6.SPINE172 29303471

[24] Choi KC Park CK. Percutaneous endoscopic lumbar discectomy for L5-S1 disc herniation: consideration of the relation between the iliac crest and L5-S1 disc. Pain Physician. (2016) 19(2):E301–8. 10.36076/ppj/2016.19.E301 26815257

[25] Liu J Wu J Zhang H Zuo R Liu J Zhang C. Application of a targeted and quantificational foraminoplasty device in percutaneous transforaminal endoscopic discectomy for L5-S1 disc herniation: preliminary clinical outcomes. J Orthop Surg Res. (2021) 16(1):398. 10.1186/s13018-021-02533-z 34158087 PMC8218444

[26] Chen Z Zhang L Dong J Xie P Liu B Wang Q Percutaneous transforaminal endoscopic discectomy compared with microendoscopic discectomy for lumbar disc herniation: 1-year results of an ongoing randomized controlled trial. J Neurosurg Spine. (2018) 28(3):300–10. 10.3171/2017.7.SPINE161434 29303469

[27] Mahmoudi Alami F Taghipour M Talebi G Sa’adat P Seyedhoseinpoor T Rad HV Comparison of lumbar muscle morphology in patients with chronic nonspecific low back pain with and without clinical lumbar segmental instability. PLoS One. (2024) 19(4):e0301726. 10.1371/journal.pone.0301726 38574091 PMC10994386

[28] Evran S Katar S. Evaluation of the effectiveness of transforaminal epidural steroid injection in far lateral lumbar disc herniations. Ideggyogy Sz. (2021) 74(1-2):27–32. 10.18071/isz.74.0027 33497058

[29] Liao Z Chen W Wang CH. Transforaminal percutaneous endoscopic surgery for far lateral lumbar intervertebral disk herniation. Orthopedics. (2014) 37(8):e717–27. 10.3928/01477447-20140728-58 25102508

[30] Sinkemani A Hong X Gao ZX Zhuang SY Jiang ZL Zhang SD Outcomes of microendoscopic discectomy and percutaneous transforaminal endoscopic discectomy for the treatment of lumbar disc herniation: a comparative retrospective study. Asian Spine J. (2015) 9(6):833–40. 10.4184/asj.2015.9.6.833 26713113 PMC4686386

[31] Wang J Xu X Wang L Huang W. Prognostic factors for patient-reported satisfaction after percutaneous lumbar endoscopic discectomy at a minimum of two years’ follow-up. Sci Rep. (2024) 14(1):22194. 10.1038/s41598-024-73366-z 39333776 PMC11436844

[32] Lattig F Fekete TF O'Riordan D Kleinstück FS Jeszenszky D Porchet F A comparison of patient and surgeon preoperative expectations of spinal surgery. Spine. (2013) 38(12):1040–8. 10.1097/BRS.0b013e318269c100 22825477

[33] McGregor AH Doré CJ Morris TP. An exploration of patients’ expectation of and satisfaction with surgical outcome. Eur Spine J. (2013) 22(12):2836–44. 10.1007/s00586-013-2971-6 23989747 PMC3843807

